# The multi-functional SNARE protein Ykt6 in autophagosomal fusion processes

**DOI:** 10.1080/15384101.2019.1580488

**Published:** 2019-03-17

**Authors:** Franziska Kriegenburg, Levent Bas, Jieqiong Gao, Christian Ungermann, Claudine Kraft

**Affiliations:** a Institute of Biochemistry and Molecular Biology, ZBMZ, Faculty of Medicine, University of Freiburg, Freiburg, Germany; b Max F. Perutz Laboratories, Vienna Biocenter, University of Vienna, Vienna, Austria; c Biochemistry Section, Department of Biology/Chemistry, University of Osnabrück, Osnabrück, Germany; d Center of Cellular Nanoanalytics Osnabrück (CellNanOs), University of Osnabrück, Osnabrück, Germany; e CIBSS - Centre for Integrative Biological Signalling Studies, University of Freiburg

**Keywords:** Autophagy, SNARE, fusion, YKT6

## Abstract

Autophagy is a degradative pathway in which cytosolic material is enwrapped within double membrane vesicles, so-called autophagosomes, and delivered to lytic organelles. SNARE (Soluble *N*-ethylmaleimide sensitive factor attachment protein receptor) proteins are key to drive membrane fusion of the autophagosome and the lytic organelles, called lysosomes in higher eukaryotes or vacuoles in plants and yeast. Therefore, the identification of functional SNARE complexes is central for understanding fusion processes and their regulation. The SNARE proteins Syntaxin 17, SNAP29 and Vamp7/VAMP8 are responsible for the fusion of autophagosomes with lysosomes in higher eukaryotes. Recent studies reported that the R-SNARE Ykt6 is an additional SNARE protein involved in autophagosome-lytic organelle fusion in yeast, *Drosophila*, and mammals. These current findings point to an evolutionarily conserved role of Ykt6 in autophagosome-related fusion events. Here, we briefly summarize the principal mechanisms of autophagosome-lytic organelle fusion, with a special focus on Ykt6 to highlight some intrinsic features of this unusual SNARE protein.

## Introduction

Eukaryotes harbor an elaborate endo-membrane system composed of organelles, which allows spatial and functional separation of cellular processes. Cell function requires communication and exchange between these organelles via membrane contact sites and intracellular vesicular trafficking [1,2]. During trafficking, vesicles form at a donor membrane and are then actively transported to the target organelle. At the target membrane fusion occurs, resulting in cargo delivery [2]. Cellular trafficking pathways include the endocytosis of extracellular material at the plasma membrane and subsequent cargo delivery to lysosomes/vacuoles via the endosomal system, and the exocytic pathway of newly synthesized macromolecules from the ER through the Golgi network to the plasma membrane or lytic organelles [3].

**Figure 1.**
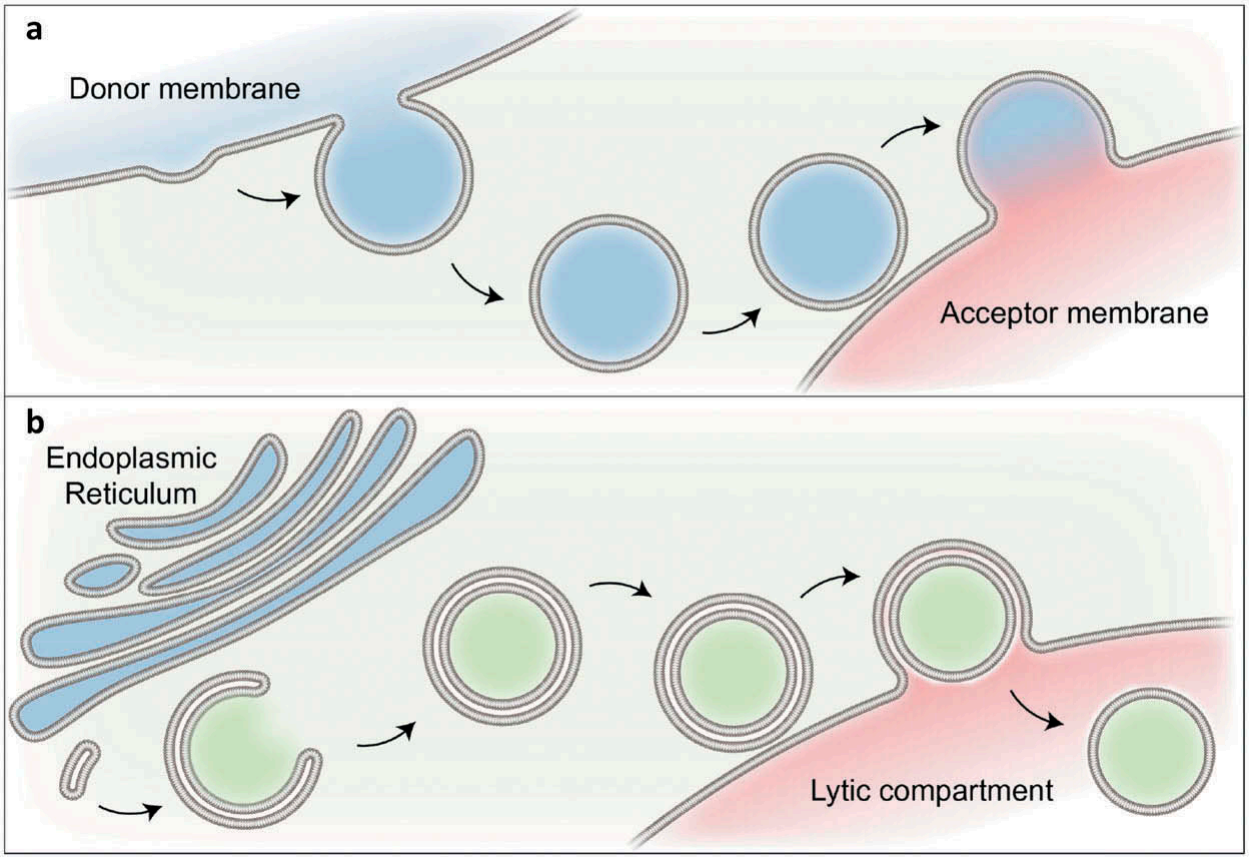
Autophagy is a unique membrane-trafficking pathway. Vesicle membrane trafficking generally starts with the deformation of a donor membrane into a bud and the nascent transport vesicle gets pinched off. The vesicle is then actively transported to a target membrane where both membranes are tethered and finally fused (A). In contrast, autophagosomes, a unique type of transport vesicle, are formed at or close to the ER. Instead of a continuous outgrowth of one membrane, the elongation of the autophagosomal precursor is thought to require the concerted action of several membrane sources/vesicles. After its closure, the emerging double membrane vesicle is transported to lytic organelles where the membranes are tethered and the outer autophagosomal membrane finally fuses with the target membrane (B).

The transport of cytosolic molecules or large intracellular structures to lysosomes/vacuoles via macroautophagy (hereafter called autophagy) represents a special trafficking event [4,5]. Unlike vesicular trafficking along the endomembrane system, whereby a small vesicle buds off a donor organelle (Figure 1(a)) [2], in autophagy a double membrane vesicle, the autophagosome, is newly formed around the cargo (Figure 1(b)) [6]. After closure of this unconventional vesicle, the engulfed material is transported to the lysosome/vacuole, where the autophagosome fuses with the lytic compartment to release the inner contents for degradation [6].

Autophagy can be distinguished as bulk autophagy or selective autophagy [5,7,8]. During bulk autophagy, cytoplasmic material is non-selectively enwrapped into autophagosomes [5,7]. The subsequent lysosomal turnover of the cargo into its building blocks allows cells to maintain energy and metabolite levels during periods of starvation [5,7]. During selective autophagy, autophagosomes engulf distinct cargo, such as pathogens or damaged and/or superfluous material, including entire organelles [8]. Thus, both types of autophagy support cell survival [4,5].

Autophagosome formation requires a highly orchestrated interplay between autophagy-related (Atg) proteins. Briefly, at the onset of autophagy in yeast, the Atg1 kinase complex activates the phosphoinositide-3-kinase Vps34 complex I to produce phosphatidylinositol-3-phosphate (PI3P) at the phagophore assembly site (PAS) [6]. The PAS is so far an ambiguous structure of proteins and membranes located near the endoplasmic reticulum (ER) and the vacuole [9,10]. Subsequent to membrane modification with PI3P, other effector Atg proteins are recruited to the PAS, which regulate the formation and expansion of the autophagosomal membrane [6]. Autophagosomal membrane elongation is promoted by (i) the covalent attachment of the ubiquitin-like protein Atg8 to the lipid phosphatidylethanolamine (PE) at the inner and outer membrane of the nascent vesicle, and (ii) the engagement of small Golgi-derived vesicles containing the transmembrane protein Atg9 [6]. Once cargo enclosure is complete and the nascent autophagosome has matured into a closed double membrane vesicle, fusion occurs between the outer autophagosomal membrane and the vacuole [6]. These general processes in autophagy are largely conserved from yeast to mammals; however, their complexity increases in higher eukaryotes [11]. For instance, metazoan autophagosomes can fuse not only with lysosomes but also with late endosomes [5,6]. For simplicity, we use the term lysosome (also with reference to the vacuole) throughout this review and specify only when needed.

Our understanding of the autophagosome-lysosome fusion process has greatly advanced over recent years. As for other vesicular fusion events, autophagosome fusion with the lysosome depends on distinct Rab GTPases, tethering factors, and a specific subset of SNARE proteins [12]. The latter constitute the minimal fusion machinery [13], whereby SNAREs from adjacent membranes assemble into a so-called *trans*-SNARE complex [2]. The energy released during their extraordinary tight interaction drives fusion of the two membranes and results in a *cis*-SNARE complex, which is dissociated to allow SNARE recycling and new rounds of fusion [2,14,15]. Tethering factors, which mostly require Rab GTPases to bind to membranes, bring membranes in contact and thus support both *trans*-SNARE assembly, but also their final fusion promoting activity [16]. Some core components of the fusion machinery are described in this review, with a focus on the SNARE Ykt6.

## 1. The Rab GTPase module

Given the variety of cellular membrane structures, it is critical that vesicles only fuse with the appropriate target membrane at the end of each trafficking event. There are likely several layers of regulation to ensure this specificity. One determinant of membrane identity includes Rab GTPases, a group of small evolutionary conserved prenyl-modified proteins that influence several aspects of membrane traffic [3,17–19]. Rab GTPases cycle between an inactive GDP-bound state in the cytosol, where they are kept soluble by GDI (GDP dissociation inhibitor) [19,20], and an active GTP-bound state at the membrane. GEFs (guanine nucleotide exchange factors) at the membrane promote the replacement of GDP to GTP within Rab GTPases [21,22], triggering a conformational change within the Rab GTPase that enables effector protein binding, localization, and function [22,23]. Many effector proteins have been identified, ranging from kinases to motor proteins, to tethering complexes and each Rab GTPase interacts with a specific subset of effectors [19,24]. Effectors selectivity, together with the control of membrane targeting and of Rab GTPase activation, provide specificity not only in fusion but also in membrane trafficking more generally [19].

Vesicular fusion with the lysosome depends on the Rab GTPase RAB7 (Ypt7 in yeast) [25,26], which is recruited to and activated at autophagosomal and lysosomal membranes by its GEF Mon1-Ccz1 [27–29]. Similar to other fusion events, the switch from the GDP- to the GTP-bound state of RAB7/Ypt7 likely needs to be spatiotemporally regulated to avoid premature fusion of immature autophagosomes with the lysosome. The GEF Mon1-Ccz1 is critical to promote fusion events, and affects RAB7/Ypt7 localization and activity [30–34]. Consequently, factors that regulate the membrane association of Mon1-Ccz1 likely have a large impact on fusion [34].

Recruitment of Mon1-Ccz1 to the autophagosomal membrane in yeast depends largely on LIR (LC3 interacting region)-mediated interactions between Ccz1 and Atg8 [29,35]. Mon1-Ccz1 binding to membrane-bound Atg8 results in increased Mon1-Ccz1 GEF activity *in vitro* [29]. PI3P has also been implicated to enhance Mon1-Ccz1 activity and to promote its association with membranes [27,36,37]. Although likely beneficial, PI3P might not be absolutely required to recruit Mon1-Ccz1, and as a consequence RAB7/Ypt7, to membranes in general [29,32]. The recruitment of Ypt7 to autophagosomal membranes, however, does depend on PI3P [29,38]. One possible scenario is that PI3P is mainly required to promote the GEF activity of Mon1-Ccz1 on autophagosomes, which results in efficient Ypt7-GTP recruitment.

On the lysosomal membrane, the mammalian RMC1 (regulator of MON1-CZZ1) binds to MON1-CCZ1 and has been suggested to stimulate its GEF activity [39]. Additional regulatory mechanisms controlling Mon1-Ccz1 and RAB7/Ypt7 on both membranes likely exist in order to regulate membrane specificity during fusion processes.

Activation of the Rab GTPase RAB7/Ypt7 is required for its binding to the effector complex HOPS (homotypic fusion and protein sorting) [40–43]. HOPS belongs to the multisubunit tethering complexes and consists of the subunits Vps11, Vps16, Vps18, Vps33, Vps39, and Vps41 [40,41]. In yeast, this heterohexameric complex is crucial for fusion events at the vacuole, because it tethers opposing membranes, proofreads SNARE complex formation, and facilitates the transition from hemifused to fully fused membranes [16,41,44,45]. HOPS tethers opposing RAB7/Ypt7-positive membranes through the Vps39 and Vps41 subunits present at opposite ends of its elongated, sea-horse like structure [46,47], though also more extended structures of HOPS have been observed [48]. Moreover, it has been suggested that phosphorylation of yeast Vps41 ensures that only membranes with GTP-bound Ypt7 are tethered, which could regulate the coordination of fusion onset [49,50]. HOPS recruitment to the fusion site and tethering is more complicated in higher eukaryotes, and involves additional factors besides RAB7, such as RAB2, ARL8B, and the RAB7 effector PLEKHM1 [51–53].

The HOPS subunit Vps33, which belongs to the SM (Sec1/Munc18) protein family, is critical for SNARE regulation [45]. SM proteins regulate different aspects of SNARE assembly, and both promoting and inhibitory functions have been observed [45,54–56]. Vps33 interacts with different SNARE domains [45,57], and together with its neighboring subunit Vps16, provides a binding platform on the HOPS surface for the R-SNARE and Q-SNAREs, thus facilitating fusion [45]. In autophagy, HOPS associates with the metazoan autophagosomal SNARE Syntaxin17 (STX17) and has been suggested to stabilize the STX17-SNAP29 SNARE assembly and/or the *trans*-SNARE complex [58,59]. HOPS might also interact with the recently identified autophagosomal SNARE Ykt6, though conflicting data exists [60–62]. In yeast, HOPS is known to associate to the vacuolar Q-SNAREs Vam3 and Vam7 [63,64] which are crucial for autophagy [38,65].

**Figure 2.**
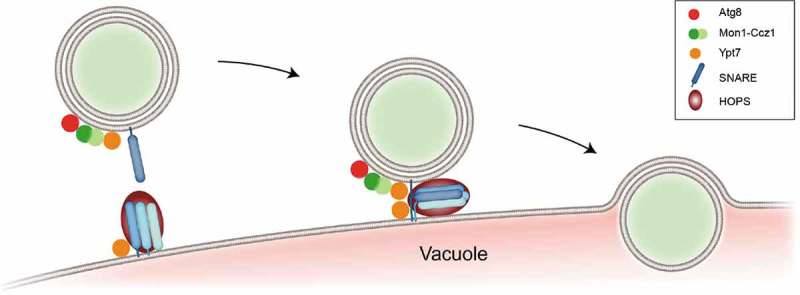
Critical components involved in the fusion of autophagosomes with lytic organelles. Prior to membrane fusion, the autophagosome and the lytic organelle (here, the yeast vacuole) are brought into close contact with the help of several factors. The PI3P-binding GEF Mon1-Ccz1 (green) associates with the autophagosomal membrane. In yeast, this is mediated via Atg8 (red). Mon1-Ccz1 stimulates the GDP-to-GTP exchange in RAB7/Ypt7 (orange). The activated RAB7/Ypt7 GTPase, present on both the autophagosome and lytic organelle subsequently recruits the tethering factor HOPS (dark red). HOPS not only bridges the opposing membranes but also influences SNARE complex formation. Final fusion of the outer autophagosomal membrane with the lytic organelle is then driven by SNARE assembly into a tight four-stranded α-helical bundle. The SNARE proteins involved in autophagosome-lytic organelle fusion are Ykt6, Vam3, Vti1 and Vam7 in yeast and STX17, SNAP29, Vamp7/VAMP8, STX7 and YKT6 in metazoan (for topology refer to main text and [75]).

Taken together, the evolutionarily conserved proteins RAB7/Ypt7, Mon1-Ccz1, and HOPS localize to both the autophagosomal and lysosomal membrane in order to coordinate SNARE-mediated fusion (Figure 2), and autophagosomes accumulate in the absence of these core components, reflecting their central role in autophagy.

## 2. The SNARE module

Membrane identity is also established by the presence of specific SNARE protein subsets on distinct membranes, where they are essential for membrane fusion.

SNARE proteins have a 60–70 amino acid long canonical SNARE domain towards their C-termini containing heptad repeats [66]. The assembly of four SNARE domains leads to the formation of a highly stable four-stranded α-helical bundle, with characteristic arginyl or glutamyl residues within the core of the structure [66,67]. SNAREs are classified into R-SNAREs or Q-SNAREs based on their contribution of either arginine (R) or glutamine (Q), respectively, to this central motif. Q-SNAREs are further classified into Q_a_, Q_b_, and Q_c_ SNAREs based on sequence homology [68,69]. In addition, Q_bc_ SNAREs exist in which one SNARE protein contains two SNARE domains [69]. The energy released during the extremely tight association between an R-SNARE from one membrane and a Q_abc_-SNARE subcomplex from the opposing membrane brings the respective lipid bilayers into direct contact, allowing a hemifused membrane intermediate to form [2,15,70,71]. This hemifused membrane intermediate subsequently resolves into its fully fused state with the help of accessory proteins such as the HOPS complex [16].

The mammalian SNARE complex for autophagosome-lysosome fusion consists of the Q_a_-SNARE STX17 and the Q_bc_-SNARE SNAP29 at the autophagosomal membrane and the R-SNARE VAMP8 at the lysosomal membrane [72]. A recent study suggests the involvement of the R-SNARE VAMP7 instead of VAMP8 at the lysosome [73]. This finding would recapitulate the SNARE complex in *Drosophila*, in which Syx17 (STX17 homolog) and ubisnap/Snap29 (SNAP29 homolog) are the functional SNAREs at the autophagosomal membrane, and the lysosomal SNARE is Vamp7 [74]. It should be noted that flies lack a VAMP8 homolog. However, given the evolutionary conservation of the Q_abc_-SNARE subcomplex from flies to mammals, the contributions of the R-SNARE VAMP7 and/or VAMP8 in the mammalian system might need further investigation. Notably, both VAMP7 and VAMP8 associate with STX17 in mammals [72], and several functional SNARE interactions might exist. Supporting this notion, an additional SNARE module harboring the R-SNARE YKT6 was recently shown to participate in autophagosome-lysosome fusion in mammals and flies [61,62,75].

## 3. Ykt6 in autophagosome-lysosome fusion

Residual autophagosome-lysosome fusion is still observed when STX17 is depleted in mammals, pointing to a second SNARE module in addition to the previously identified STX17, SNAP29, VAMP8 SNAREs [61,72,76]. To identify the alternative mammalian SNARE(s) involved, Matsui et al. (2018) analyzed autophagosomes that accumulated fusion-impaired cells *in vivo* and detected the SNARE protein YKT6. Immunofluorescence and flotation studies revealed that YKT6 localizes to late autophagosomal structures [61]. Time-controlled depletion of *YKT6* was found to impair autophagosome-lysosome fusion without affecting other lysosomal functions tested. Further, simultaneous depletion of *STX17* and *YKT6* abolished autophagosome-lysosome fusion completely [61]. YKT6 shows promiscuous binding to nearly all mammalian Q_a_-SNAREs, preventing the straightforward identification of its Q_abc_ SNARE partners at the lysosome [61]. The lysosomal Q_a_-SNARE STX7 (syntaxin 7) was known to be involved in lysosomal fusion events with endosomes [77,78], and depletion of *STX7* resulted in impaired autophagy, similar to *YKT6* and *STX17* [61]. Moreover, loss of both *STX17* and *STX7* showed additive defects on autophagosomal fusion whereas simultaneous knockdown of *STX7* and *YKT6* did not, suggesting that STX7 and YKT6 act in one pathway. YKT6 and STX7 were shown to interact *in vivo*, and this interaction was enhanced by overexpressing SNAP29. These data led to the conclusion that YKT6, together with STX7 and SNAP29, act as a second functional SNARE complex in addition to STX17, SNAP29 and VAMP8 during autophagosome-lysosome fusion [61].

In contrast to YKT6 in mammals, Ykt6 in *Drosophila melanogaster* seems to localize to lysosomal structures and does not appear to act in parallel to the Syx17/Snap29/Vamp7 SNARE complex [62]. Rather, it has been suggested that Ykt6 regulates their *trans*-SNARE assembly, whereby the lysosomal Ykt6 precedes Vamp7 binding to the autophagosomal Q_abc_-SNARE sub-complex Syx17/Snap29 in order to allow efficient HOPS recruitment [62]. Deletion of *Ykt6* in *Drosophila melanogaster* phenocopies the complete autophagosome-lysosome fusion defect observed after loss of Syx17, Snap29, Vamp7 or HOPS [59,62,74]. This finding, and the observation that Ykt6 localizes to lysosomal structures, suggested that Ykt6 might act as an alternative SNARE for Vamp7. However, *in vivo* and *in vitro* binding studies revealed that *Dm*Ykt6 associates only weakly with the Syx17/Snap29 SNARE subcomplex, and the interaction could be outcompeted by Vamp7 [62]. The unusually weak interaction of the SNARE domains of Ykt6, Syx17 and Snap29 was proposed to reflect steric hindrance within the formed SNARE bundle. In addition, mutation of the central arginyl residue in Ykt6 does not affect autophagy [62]. The findings that Ykt6 does not form a tight and functional SNARE bundle with Syx17/Snap29, but that its loss completely blocks autophagy, led to the proposal that it plays a regulatory role in SNARE formation [62]. Because *Dm*Ykt6 can bind HOPS *in vitro* [62], it was suggested that Ykt6 facilitates HOPS recruitment to the autophagosome-lysosome fusion site, which is followed by Ykt6 replacement with Vamp7 [62].

The apparent difference in Ykt6 function is surprising but might not be mutually exclusive. It is conceivable that Ykt6 might act as a genuine autophagosomal SNARE in autophagosome-lysosome fusion in the germline in flies. In line with this notion, *Drosophila* Ykt6 also co-localizes with the autophagosomal marker Atg8, and it likely binds to Snap29 [62]. Conversely, mammalian YKT6 is also found on lysosomes, which could result from *cis*-SNARE complex formation but might also point to a regulatory role for YKT6 at the lysosomal membrane in mammals [61]. How the roles for Ykt6 in autophagosome-lysosome fusion compare and contrast in *Drosophila* and mammals needs further investigation.

Recent work has also illuminated the role of Ykt6 during autophagosome-vacuole fusion in yeast [38,65,75]. To address the mechanism of autophagosome-vacuole fusion in yeast, we established *in vitro* reconstitution assays. We used fluorescence microscopy to monitor the fusion of isolated vacuoles and enriched autophagosomal fractions. The direct observation of fusion allowed us to elucidate the components required for fusion and their regulation. Our study found that the Rab GTPase Ypt7 at both vacuoles and autophagosomes is crucial for autophagosome-vacuole fusion. We showed that Ypt7 recruitment to the autophagosome depends on (i) PI3P formation by the PI3-kinase complex I and (ii) Mon1-Ccz1 [29,38,65]. The Ypt7 effector complex HOPS is also indispensable for autophagosome-vacuole fusion, and is required at the vacuolar site to stimulate fusion [29,38,65]. Using the cell-free *in vitro* reconstitution assays further allowed us to overcome previous limitations and define the involvement and exact topology of the specific, essential SNARE proteins. The SNAREs Vam3, Vam7, Vti1 and Ykt6 have previously been shown to affect autophagy in yeast [79–83]. Whereas mutant variants of the Q-SNAREs Vam3 and Vti1 on isolated vacuoles blocked autophagosome-vacuolar fusion, mutant R-SNARE Ykt6 on enriched autophagosomes impaired fusion [38,65]. Mutant variants of other tested SNARE proteins showed no effect. The use of antibodies against these SNAREs further confirmed the necessity of Vam3, Vam7, Vti1 and Ykt6 [65]. Besides Ykt6, none of the other metazoan SNAREs that mediate autophagosomal fusion have homologs in yeast. The high degree of functional conservation of Ykt6 between yeast and mammals points to a central role for this SNARE in autophagosome-lysosome fusion. Below, we highlight some of the critical features of Ykt6.

## 4. The SNARE Ykt6

The evolutionarily conserved R-SNARE Ykt6 was first identified in yeast, in complex with Sed5 [84,85], a Q_a_-SNARE involved in vesicular trafficking between the ER and *cis*-Golgi. Yeast depleted of Ykt6 showed a block in the early stages of the exocytic pathway [85], confirming the role of Ykt6 in Sed5-dependent membrane trafficking, where Ykt6 mainly mediates retrograde trafficking from the *cis*-Golgi to the ER [85,86]. Further studies, using various Ykt6 mutants in yeast, connected Ykt6 to several other trafficking events, including late steps during both protein secretion and protein transport to the vacuole [82,86]. The observation that Ykt6 disruption similarly impairs functionally distinct transporting routes to the vacuole, such as CPY (carboxypeptidase Y) shuttling from the ER through the Golgi or Ape1 transport during selective autophagy, lead to the assumption that Ykt6 is involved in vesicular membrane fusion at the vacuole [86]. Many studies support a role for Ykt6 as a multifunctional SNARE in different intracellular membrane trafficking events at the Golgi, endosome, autophagosome, and vacuole [38,61,82,86–88]. Therefore, not surprisingly, loss of Ykt6 is lethal in eukaryotes [62,85].

**Figure 3.**
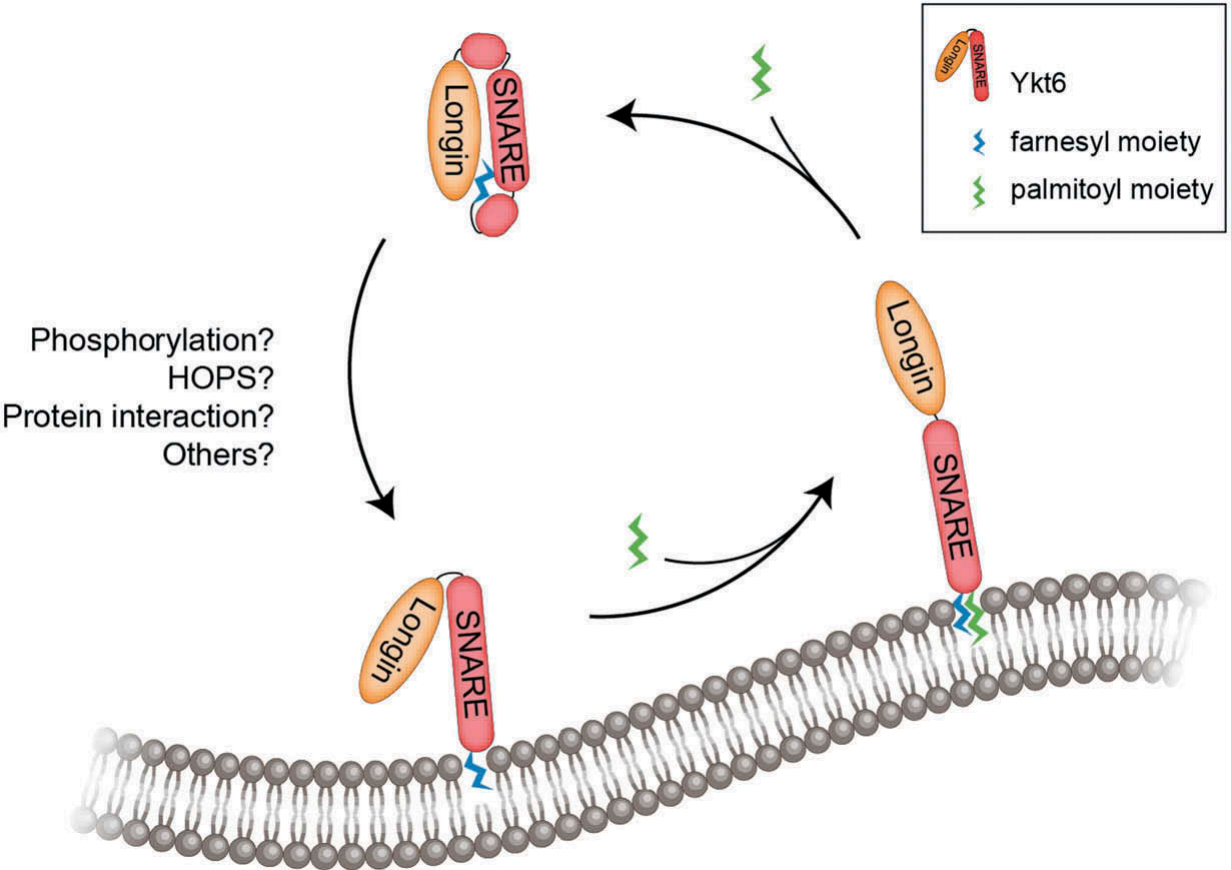
Ykt6 cycles between a cytosolic and membrane-bound state. The SNARE protein Ykt6 consists of an N-terminal longin domain (orange), a C-terminal SNARE domain (red) and a C-terminal lipidation site, which is post-translationally farnesylated (blue) and can furthermore be reversibly palmitoylated (green). In the cytosol, Ykt6 is present in a closed and inactive conformation, in which the N-terminus of Ykt6 is folded back and tightly interacts with the partially folded SNARE domain. The interaction creates an internal hydrophobic core where the farnesyl-moiety is engaged and shielded from the cytosol, keeping Ykt6 soluble and further stabilizing the closed state. Upon a so-far-unknown trigger (potentially protein-protein interactions, i.e. with HOPS, and/or phosphorylation), which possibly causes a change to a more open conformation, Ykt6 is recruited to membranes where it is membrane anchored through the integration of the farnesyl residue into the lipid-bilayer. Ykt6 membrane localization is further stabilized by palmitoylation. Ykt6 can be released again from membranes, which coincides with de-palmitoylation and re-adopting its closed conformation.

The manifold functions of Ykt6 might be reflected by its promiscuous interactions with Q-SNAREs [61,89], and its flexibility in membrane localization. In contrast to most SNAREs, which harbor a transmembrane domain next to their C-terminal SNARE domain, Ykt6 possesses a C-terminal prenylation consensus site (CAAX motif) with a preceding cysteinyl residue [85]. Both cysteines are highly conserved, and each can be lipid-modified through reversible palmitoylation on the first and permanent farnesylation on the second cysteinyl residue [88,90]. Depending on its lipid state, Ykt6 can cycle between the cytosol and membranes, and this is greatly influenced by its N-terminal longin domain (Figure 3) [91]. Longin domains form five antiparallel β-strands sandwiched between two α-helices on one side and a single α-helix on the other [92]. This domain was first characterized in SNARE proteins only, but was subsequently found in seven superfamilies, all of which are involved in membrane trafficking events, such as Mon1 and Ccz1 [92]. The longin domain of Ykt6 affects its membrane targeting and SNARE activity [91,93]. In its cytosolic state, Ykt6 adopts a highly compact and stable fold, in which its N-terminal longin domain assumes a strong intramolecular interaction with the C-terminal SNARE domain, mediated through a hydrophobic patch present in the N-terminus [91,94]. The farnesyl-moiety is engaged and shielded from the cytosolic environment within the hydrophobic core of the fold, which keeps Ykt6 in a soluble state and further stabilizes the closed and fusion-inactive confirmation [94,95]. Similar autoinhibition mechanisms, in which the N-terminal domain folds back and interacts with the SNARE domain, have been observed for other SNARE proteins [54]. Syntaxins (Q_a_-SNAREs) contain an N-terminal domain, which consists of a three α-helical bundle (H_abc_) [96], that can flip back onto the SNARE core and result in a closed confirmation [55,97]. In addition to Ykt6, VAMP7 harbors a longin domain and similarly adopts a locked fold [92].

The longin domain of Ykt6 has been suggested to at least partially mediate membrane targeting [61,93,98]; however, the precise mechanism underlying how Ykt6 localizes to several distinct membrane sources is unknown. Various point mutations within the longin domain of Ykt6 destabilize the intramolecular interaction between the longin and SNARE domain, which results in a more open conformation accompanied by relocalization of Ykt6 from the cytosol to membrane structures [91,93]. These data suggest that liberation of the farnesyl-moiety from the hydrophobic center allows Ykt6 to integrate into membranes. It is tempting to speculate that protein-protein interactions with, or modifications of, the Ykt6 N-terminus trigger a conformational change from a closed to an open state. In the case of syntaxins, the interaction of SM proteins with the N-terminal H_abc_-domain lifts the inhibitory state [97]. The H_abc_-domain of the autophagosomal SNARE STX17 interacts with the HOPS SM subunit VPS33A and it has been suggested VPS33A stabilizes both the closed conformation of STX17, as well as its open form complexed with other SNAREs [73,99]. This regulation seems to depend on the phosphorylation status of the STX17 N-terminus [73]. A phosphorylation-dependent switch from a closed to an open state has also been proposed for the longin domain of VAMP7 [100]. It will be interesting to elucidate if similar or novel mechanisms regulate the conformational change in Ykt6, during its cycling from the cytosol to membranes. Interestingly, the longin domain of Ykt6 from flies and yeast Ykt6 can interact with HOPS *in vitro* [60,62], though conflicting data exist, and no association between Ykt6 and HOPS could be detected in mammals [61]. Reconstitution of autophagosome-vacuolar fusion depends on ATP, which is needed to disassemble the tight four α-helical bundled SNARE complex after fusion [14,38,65,101]. Although speculative, we propose that the observed ATP-dependency could in part reflect regulatory protein phosphorylation events, which could include Ykt6.

After primary membrane association of Ykt6 via its farnesyl-moiety, Ykt6 is subsequently palmitoylated by cellular DHHC palmitoyl-transferases [98,102], possibly supported by intrinsic palmitoylation activity [103,104]. The palmitoylation of Ykt6 further stabilizes its membrane-bound state [90]. The loss of membrane localization after deleting or mutating both conserved cysteines of the extended CAAX motif is lethal and shows that Ykt6 function requires membrane integration [62,85,88,90]. After each fusion event, the resulting *cis*-SNARE complex needs to be disassembled by the action of the ATPase *N*-ethylmaleimide-sensitive factor (NSF), Sec18 in yeast, and its co-factor α-SNAP (Sec17 in yeast) [14,101]. The disentanglement of the *cis*-SNARE complex occurs along with the release of non-palmitoylated Ykt6 from the membrane [88,98], which may indicate a coordinated Ykt6 de-palmitoylation step during NSF-dependent SNARE disassembly.

Owing to their ambiguous formation, it is unclear when and how autophagosomes are equipped with SNAREs. Vesicles that emerge by budding from a SNARE-containing donor-membrane obtain their SNARE proteins already during formation. Autophagosome formation has been proposed to occur via an outgrowth of a precursor structure from the ER [105,106], the fusion of organelle-derived vesicles [6,107–110], or completely *de novo* [111]. Thus, both the ER as well as vesicles could theoretically supply the autophagosomal membrane with the SNARE proteins (Ykt6 or STX17) needed for fusion with lysosomes. However, STX17 has been reported to join autophagosomes only late during their maturation state, shortly before or during autophagosome closure [112]. STX17 is anchored to the autophagosomal membrane by an unusual tandem transmembrane structure, in which two transmembrane domains adopt a closely packed hairpin-like conformation mediated by a glycine-zipper-like motif present in each of the transmembrane helices [113]. Despite being a transmembrane protein, STX17 has been found to equally localize both to the cytosol and at membranes [72]. The capability to extract the SNARE from membranes was attributed to the low hydrophobicity of the tandem transmembrane domain. Within the cytosol, STX17 may be kept soluble by specific protein-protein interactions. IRGM (immunity related GTPase M) has been proposed to bring STX17 to the autophagosomal membrane [114], however, additional protein(s) likely bind to the cytosolic pool of STX17, since the IRGM/STX17 complex is mainly membrane-associated [114]. Furthermore, the Q_bc_-SNARE SNAP29, which forms the Q_abc_ subcomplex with STX17 [72,74], is a cytosolic protein and lacks any kind of membrane-anchor [115]. This allows SNAP29 to transiently associate with membranes [116]. Together, these findings explain how SNARE proteins can be recruited to already mature autophagosomes. The ability of Ykt6 to diffuse freely in the cytosol in a closed and inert state suggests that Ykt6 might also join autophagosomes only once they have matured.

Why mammals have two SNARE modules is unknown. Given that loss of STX17 and YKT6 in mammals has additive detrimental effects on autophagosome-lysosome fusion [61], one possible scenario is that two populations of autophagosomes exist, with either YKT6 or STX17 at their surface. The STX17 SNARE module is involved in starvation induced bulk autophagy [72,74], but has no effect on tested selective-types of autophagy [76]. One might therefore speculate that in the mammalian system, STX17 primarily mediates fusion of larger bulk autophagosomes, whereas YKT6 could act on smaller selective autophagosomes. Notably, autophagosomes from yeast are on average smaller than their metazoan counterparts [117]. With regard to the conservation of Ykt6, the lipid-anchor of Ykt6 could be better suited for the smaller autophagosomes with higher membrane curvature, which could include mammalian selective-type autophagosomes and yeast autophagosomes. The tandem transmembrane domain of STX17, on the other hand, might preferably integrate into larger autophagosomes with lower membrane curvature, such as mammalian bulk autophagosomes. Another hypothesis to explain the presence of both YKT6 and STX17 is based on the capacity of metazoan autophagosomes to fuse with different target membranes. Thus, it has been proposed that YKT6 and STX17 provide the selectivity for fusion with either lysosomes or late endosomes [61].

In conclusion, the structural features of Ykt6 allow this SNARE to be freely mobile and flexibly integrated into various membrane sources, which might be especially critical at the autophagosomal membrane. Moreover, Ykt6 can interact with multiple SNAREs, and its promiscuity for SNAREs and membranes makes it a versatile SNARE in membrane fusion events.

## Concluding remarks

The unconventional membrane association mechanism of Ykt6 in yeast and mammals provokes the speculation that Ykt6, similar to STX17, joins autophagosomes only once they have matured. Whether Ykt6 in *Drosophila* plays only a regulatory role or serves additional functions during fusion are interesting topics for upcoming research.

Several questions remain unanswered in the field, surrounding how fusion of opposing membrane sources is coordinated and synchronized: When and how is Ykt6 recruited and activated at the autophagosomal membrane? How does the SNARE domain of Ykt6 differ from other less promiscuous SNAREs? When and how is Mon1-Ccz1 recruited? Is the requirement of Atg8 for Mon1-Ccz1 recruitment conserved in higher eukaryotes? What is the effect of HOPS on the single SNAREs during the fusion process? Addressing these questions will develop our understanding of autophagy and broaden our insight into cell biology more generally.
